# Editorial for the Special Issue “Preparation and Application of Advanced Functional Membranes”

**DOI:** 10.3390/membranes14050100

**Published:** 2024-04-26

**Authors:** Annarosa Gugliuzza, Cristiana Boi

**Affiliations:** 1 Institute on Membrane Technology-National Research Council, CNR-ITM, Via Pietro Bucci 17C, 87036 Rende, Italy; 2 Department of Civil, Chemical, Environmental and Materials Engineering, Alma Mater Studiorum, University of Bologna, Via Terracini 28, 40131 Bologna, Italy; 3 Department of Chemical and Biomolecular Engineering, North Carolina State University, Raleigh, NC 27695-7905, USA

## 1. Introduction

Membrane science is a discipline that cuts across almost all fields of research and experimentation [1]. It is well suited to the demand for best practices that provide sustainable solutions for natural resource management [2,3], pollution remediation [4,5], the recovery and recycling of value-added products from waste [6], and energy production and storage [7]. Advanced technologies are also being proposed for sustainable agriculture and industrial practices [8], textiles [9], biomedicine [10], packaging [11], and cultural assets [12,13]. In each case, membrane science provides multidisciplinary approaches to understand and control phenomena at different length scales, so the design and development of membrane devices and technologies requires the interplay of complementary disciplines, including modeling and simulation, membrane fabrication and characterization, process implementation, the build up of prototyping, and technology transfer. Membrane technology also has the advantage of being easy to integrate with other more conventional technologies, being remotely controllable and adaptable to different volumetric spaces [14]. Additionally, its sustainability offers the opportunity to be powered by green energy as an alternative fuel source [15]. Membrane technology is built upon both challenging and basic concepts such as the development of interfaces enabling the selective passage of bioactive molecules, ions, liquids (such as water), and gases according to well-established transport mechanisms. The latter can be regarded as the result of the intricate channels, pathways, and pores generated inside the membranes [1], as well as the accessibility to chemical moieties working as temporary adsorption sites or donor–bridge–acceptor systems [16,17].

Combining sustainable membrane design and technology is a major challenge in membrane science [18,19]. The aim is to provide solutions and best practices to manage and recover resources according to the principles of the circular green economy and durability [20,21]. Accordingly, a multidisciplinary approach is often proposed to implement the desired membrane operation on an industrial scale through process optimization, which involves a step-by-step experimental investigation and theoretical analysis, along with feasibility and cost-effectiveness assessments. First of all, the development of membranes with specific morphological and chemical features allows for the manipulation of desired or undesired events conceivable at the nano and micro scale so that the corresponding effects can be amplified at the macro scale. Once the type of operation is identified, the choice of the fabrication process that will be used to obtain suitable structural and chemical features in a predefined volumetric space becomes crucial. In fact, a jigsaw puzzle of functions can generate specific relationships for cooperative mechanisms that allow one to obtain the targets of a specific separation process. In recent years, advanced manufacturing processes, along with diverse families of materials, have been proposed to shift the perspective of membranes from traditional physical barriers [1] to interactive [22] and, where required, smart selective interfaces [23]. Thus, traditional phase inversion [1], track-etching [24], stretching [25], and interfacial polymerization [26] have been optimized or combined with more advanced fabrication methods, including phase separation micromolding [27], electrospinning [28], copolymer self-assembly [29], breath figure self-assembly [30], lithography [31], layer-by-layer assembly [32], and many others. On the other hand, different classes of materials, including organic fillers [33,34], nanotubes [35,36], nanoparticles [37,38], 2D materials [39,40], and quantum dots [41], have recently been proposed to promote (i) the assisted transport of gas/vapors [42,43], liquids [44,45], and ions [46,47] through membranes; (ii) improved selectivity [48,49] and high recovery factors [50,51]; (iii) energy saving [52,53]; and (iv) long-term durability [54,55]. 

**Figure 1:**
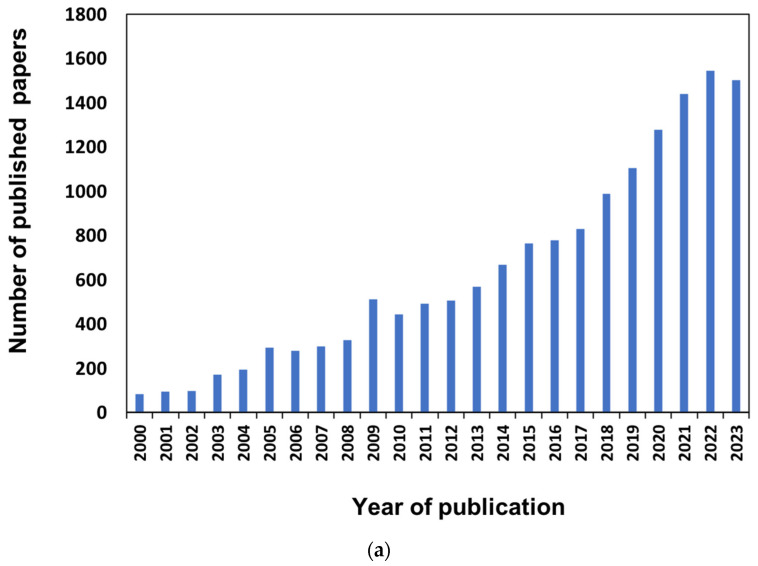

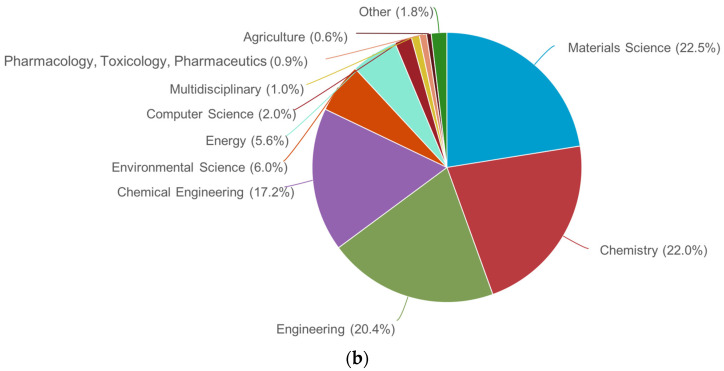
Annual publications on the subject of membrane fabrication (**a**) and subject areas’ contributions to the literature (**b**) over the past 20 years (2000–2023). Source: Scopus.

In the last two decades, the number of papers focusing on membrane fabrication has increased exponentially (Figure 1a), confirming the growing awareness of the need to develop new functional membranes for effective operations in different subject areas (Figure 1b). 

**Figure 2:**
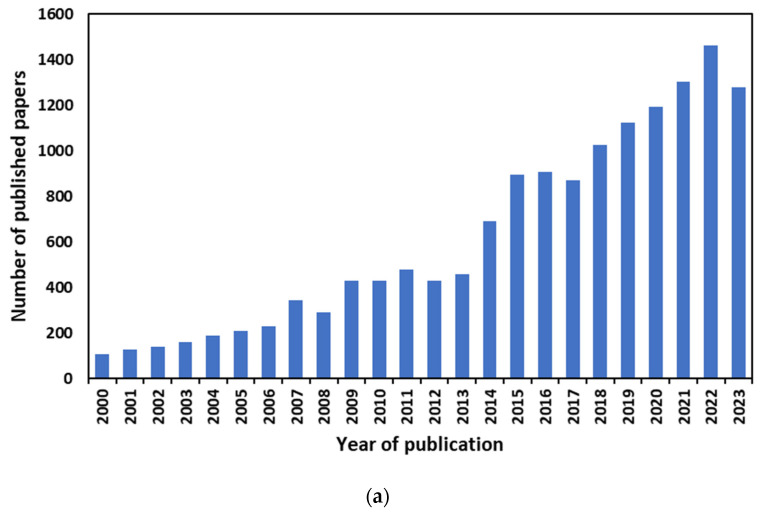

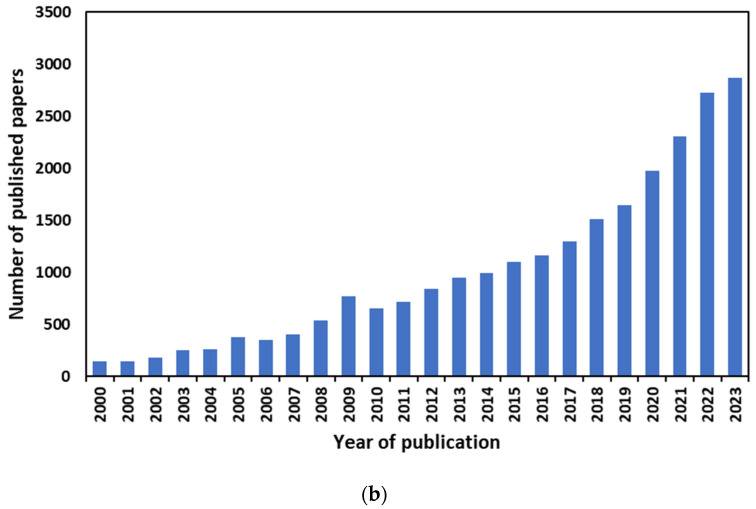
Annual publications on (**a**) membranes for water purification and (**b**) membranes for energy applications in the last 20 years (2000–2023). Source: Scopus.

At the same time, the use of membrane technology has expanded, as it is seen as a practical way to manage natural resources and provide tangible solutions for environmental remediation, including water purification, CO_2_ capture, and pollution control. For example, the number of articles on water purification in the last 20 years (Figure 2a) has shown an increasing trend, reaching a maximum of almost 1464 in 2022. The rise in the number of publications dedicated to membranes for energy applications has been exponential, reaching almost 3000 articles in 2023 (Figure 2b). This is because membranes can play a fundamental role in the more efficient use of natural resources for energy applications, with membranes suitable for application in batteries, hydrogen separation, and fuel cells serving as important examples.

This Special Issue, “*Preparation and Application of Advanced Functional Membranes*”, offers some significant novel insights into the vast research dedicated to membrane manufacturing and applications, comprising ten interesting original articles and two reviews. 

## 2. Overview of Published Articles

The ten original articles featured in this Special Issue deal with various issues related to water desalination (1), aqueous stream purification (2), antibacterial activity (1), controlled release (1), gas separation (2), the modeling of molecular transport through membranes (2), and nanohole formation in membranes (1). Two noteworthy reviews complete the Special Issue: the first focuses on nanocellulose green membranes and their related applications, and the second discusses the use of membranes for thorium removal, recovery, and recycling. Di Luca et al. [56] propose polyvinylidene fluoride (PVDF) membranes engineered with supramolecular complexes based on smart poly(N-isopropyl acrylamide) (PNIPAM) mixed hydrogels with aliquots of ZrO(O_2_C-C_10_H_6_-CO_2_) (MIL-140) and graphene to make water desalination more sustainable and effective under relatively soft working conditions. Cooperative mechanisms take place at subnanometer scale to increase freshwater production, contrast fouling events, and facilitate in situ cleaning so that distinct effects can be induced by a simple switch of the density charge at the membrane interface without the need for additional chemicals or processing steps. Regarding the results, fluxes five times higher than pristine PVDF membrane were obtained after engineered membranes coming into contact with a mixture of NaCl (35 g L^−1^) and humic acid (1 mg mL^−1^); effective antifouling and safer and more sustainable self-cleaning actions helped to (i) limit the decline in the flux with time and (ii) allow for the recovery of up to 99% of the water permeation properties of the functional membranes.

Butylskii et al. [57] developed a new recycling method for lithium-ion batteries (LIBs) using a hybrid electrobaromembrane (EBM) approach. This technique selectively separates Li^+^ and Co^2+^ ions using a special membrane with a pore diameter of 35 nm. The process combines an electric field and a pressure field to achieve highly efficient ion separation. The flux of lithium through the membrane is approximately 0.3 mol m^−2^ h^−1^, and the presence of coexisting nickel ions does not interfere with lithium separation. Also, under conditions where the feeding and receiving solutions are identical, the flux of cobalt ions can be directed from the receiving to the feeding solution, leading to a result that is close to zero (–0.0025 mol m^−2^ h^−1^) and an ion separation coefficient of Li^+^ and Co^2+^ ions of −55. EBM conditions can be tailored to extract only lithium while leaving cobalt and nickel in the feed solution with a separation coefficient equal to infinity. This process holds promise for meeting the global demand for lithium and mitigating e-waste issues.

Joosten et al. [58] developed electrospun wire membrane adsorbers based on sulfonated poly(ether-ether-ketone) (sPEEK) for the recovery of valuable resources. These membrane adsorbers selectively bind lysozyme and have potential applications in the removal of heavy metals, dyes, and pharmaceutical components. Variations in fiber diameter have minimal effect on specific surface area and dynamic adsorption capacity. Different degrees of sulfonation of sPEEK do not proportionally affect the adsorption capacity. A dynamic lysozyme adsorption capacity of 59.3 mg/g can be achieved at 10% breakthrough regardless of flow rate. This study highlights the role of fiber diameter and functional group density in optimizing membrane adsorber performance. More specifically, variations in fiber diameter and functional group density do not significantly affect the binding capacity, meaning that a membrane absorber can be realized easily and used immediately to bind positively charged molecules without further morphological and chemical adjustments. This research article provides a valuable and prompt solution for efficient resource recovery and purification.

Composite ultrafiltration membranes have been prepared by Figuereido et al. [59] using cellulose acetate and silver nanoparticles. Varying the membrane structure results in different antibacterial effects against *E. coli*. The presence of silver nanoparticles significantly inhibits the growth of *E. coli*. More permeable membranes have higher silver contents and superior growth inhibition. When fully immersed in an *E. coli* suspension, the antibacterial activity of both silver-free and AgNP membranes are suitably correlated with surface chemical composition and silver accessibility, resulting in an efficient inhibition pattern for membranes with AgNPs. These results point towards potential applications in water purification and other areas related to antibacterial action.

The research article authored by Pereira et al. [60] focuses on magnetic-responsive hydrogels for liposomal drug delivery. They have developed systems with liposomes containing ferulic acid (FA) encapsulated in gelatin hydrogel membranes with iron oxide nanoparticles (MNPs). Their study compared these systems with conventional drug delivery methods. FA release from the liposomal gelatin followed the Korsmeyer–Peppas model, suggesting controlled diffusion in the absence of a magnetic field. However, under magnetic stimulation, due to the dispersed MNPs in the matrix, the release of FA from the liposomal gelatin membrane undergoes an increase in the constant rates; while keeping a diffusional controlled FA release mechanism, the FA release changes from Fickian diffusion to quasi-Fickian diffusion. Low-intensity magnetic fields stimulate FA release and shift the mechanism. The liposomal gelatin systems therefore offer a smoother and more controlled release of FA, with the potential for longer-term therapeutic use. This approach opens doors for novel magnetically controlled drug delivery approaches in biomedicine.

Liu et al.’s article [61] presents a computational mechanistic study that was conducted to understand how to remove acid gases from natural gas by using new functionalized GO membranes. They studied the adsorption and diffusion of several gases in 1,4-phenylenediamine-2-sulfonate (PDASA)-doped GO membrane channels, providing new insights into the solubility coefficient of CO_2_ and H_2_S. They estimated, for these two gases, a binding affinity that is higher than that computed for CH_4_ and N_2_. Their theoretical analysis suggested multilayer adsorption in functionalized GO membrane channels according to the Redlich–Peterson model, while the Langmuir model was used to describe weak adsorption for no polar gases. The result is an enhanced permeability and selectivity of acid gases such as CO_2_ and H_2_S—i.e., PCO_2_ = 7265.5 Barrer, αCO_2_/CH_4_ = 95.7; P(H_2_S + CO_2_) = 42,075.1 Barrer, αH_2_S/CH_4_ = 243.8—over CH_4_, with a performance superior to that observed for more traditional GO membranes.

Polak et al. [62] provide an in depth-study of the transport properties of Pebax@2533 membranes filled with SiO_2_, ZIF−8, and POSS-Ph over a broad range of temperatures and pressures, providing permeability, diffusivity, and solubility data for N_2_, CH_4_, and CO_2_ gases. CO_2_ selectivity was also examined as a function of temperature and pressure effects. The permeability increased with temperature, leading to a predominance of the diffusivity mechanism over the solubility one. Instead, the increase in pressure enhances CO_2_ permeability, while greater benefits can be obtained for N_2_ and CH_4_ gases in terms of diffusivity.

Water vapor transport was studied through Pebax@2533 membranes filled with the ionic liquid [C_12_C_1_im]Cl (30 to 70 wt.%) by the authors of [63]. This theoretical study, which was inspired by prior experimental evidence, was conducted to establish how the addition of ionic liquid (IL) to the elastomeric membranes generates preferential pathways for water molecules, reaching values of water permeability of 85 × 10^−3^ g m^−2^ day^−1^ m at 318 K and with a content of [C_12_C_1im_]Cl around 70 wt.%. Molecular dynamics simulations yield new insights into the role of the anions and cationic head groups of IL in directing water molecule diffusion. This pathway becomes wider with increases in temperature and IL concentration due to the larger water-accessible area. The use of smart organic nanofillers such as ionic liquids is herein demonstrated to make Pebax membranes breathable to a desired extent and in a reversible way. This type of engineered membrane is extremely interesting for the construction of environmental micro-regulation devices. 

The study contributed by Strzelewicz et al. [64] explores particle diffusion in heterogeneous membrane-like structures by studying the interplay between membrane structure, external drift forces, and diffusion characteristics. They used Cauchy flight diffusion with drift and compared it to Gaussian random walk. The results show that strong drift can halt Gaussian diffusion while promoting superdiffusion with Cauchy flight. The membrane structures, which mimic real polymeric membranes with inorganic powders and designed obstacles, influence the transport behavior. More specifically, this paper proves that, under weak drift, the diffusion is controlled by the local environment, that is, the membrane. For stronger drift, superdiffusion is recognized, and in cases of excessively strong drift, the Brownian motion is almost stopped while tracers are pushed against obstacles. However, this pushing is overcome by random motion. Understanding these relationships is critical to improving the efficiency of processes that rely on membrane-based transport. This study underscores the role of structure and drift in shaping particle movement across membranes.

Ceccio et al. [65] propose the use of non-destructive ion transmission spectroscopy (ITS) and neutron depth profiling (NDP) to analyze, monitor, and quantify the evolution of etched micron-sized pores, the shape of which, in their study, transferred from latent tracks to a conical form (for one-sided etching) and, through a symmetrical (double-sided) etching process, to a well-developed cylindrical geometry. Using a traditional wet chemical etching process, they used dopants such as LiCl solution or boron to fill and generate defined pores and evaluated the effects on their shape and depth. As an example, the volume of the pore is demonstrated to increase linearly with both the etching temperature and time. Doping the etched tracks with 5 M LiCl, reductions of 24% and 11% can be obtained for the pore volume using etching times of 45 and 60 min, respectively. No destructive techniques allow for the investigation and quantification of every single effect of the working parameters on the final pore shape and size. This contribution falls into the list of studies dedicated to the fabrication and characterization of membranes with well controlled morphologies.

Nitodas et al. [66] focus on the sustainable use of nanocellulose to prepare membranes useful for a large range of applications, including water purification, desalination, antimicrobial applications, gas separation, and gas barrier applications. After an introduction to the synthesis and characterization of nanomaterials for nanocellulose membranes, case studies are illustrated through comparative analyses, highlighting the biodegradability, non-toxicity, low density, thermal stability, long-term reinforcement capabilities, and high mechanical strength of the proposed materials. Interestingly, this review provides further insights into the non-conventional uses of nanocellulose membranes in biomedical fields and other, more recreational practices, such as musical instruments.

Man et al. [67] discusses the use of membrane processes to address the removal, recovery, and recycling of thorium from industrial residues that often end up in municipal waste treatment facilities. They cover the biomedically relevant aspects of thorium, thorium detection techniques, classical extraction methods, and various membrane processes, such as electrodialysis and the use of liquid membranes. Special emphasis is placed on urban mining and the valorization of thorium. Moreover, this paper aims to provide insight into the safe handling of thorium and environmental protection through proposing practices and actions for the removal, recovery, and valorization of thorium.

## 3. Conclusions

The articles published in this Special Issue represent valuable contributions to the research dedicated to the development of new functional membranes for sustainable separation processes. Best practices are proposed to improve the performance of various separation processes, including water desalination, waste purification, CO_2_ capture, antimicrobial applications, and smart drug delivery. Conceivable solutions are anticipated to make the operation of each membrane more ecofriendly through the selection of non-polluting, recyclable, and biodegradable materials, the use of low-environmental-impact manufacturing schemes, and the implementation of purification technologies for specific case studies. It is important to note that the output of each process is always related to the specific features of the membranes, including their morphological properties and chemical functionalities, which can be manipulated by external triggers. On the other hand, the understanding of transport mechanisms is always related to the intrinsic structural and chemical aspects of the membranes, as ascertained through the carrying out of theoretical analyses and experimental research.

As a final note, we would like to highlight that this Special Issue collects transversal and complementary contributions from China, the USA, and multiple European Countries, representing a shared and enhanced cross-technology research effort based upon issues regarding environmental and health protection.
